# The iSTOPP study: Protocol for a proof-of-concept randomised clinical trial of sensory discrimination training in people with persistent neck pain

**DOI:** 10.1016/j.conctc.2021.100820

**Published:** 2021-07-14

**Authors:** Daniel S. Harvie, Nick Olthof, Andrea Hams, Hayley Thomson, Michel W. Coppieters

**Affiliations:** a Menzies Health Institute Queensland, Griffith University, Brisbane and Gold Coast, Australia; b School of Allied Health Sciences and Social Work, Griffith University, Brisbane and Gold Coast, Australia; c Gold Coast University Hospital, Gold Coast Hospital and Health Service, Gold Coast, Australia; d Amsterdam Movement Sciences, Faculty of Behavioural and Movement Sciences, Vrije Universiteit Amsterdam, Amsterdam, the Netherlands

**Keywords:** Neck pain, Chronic pain, Tactile acuity, Cortical reorganisation, Sensory training, Two-point discrimination

## Abstract

**Background:**

Neck pain can be associated with a reduction in tactile acuity that is thought to reflect disrupted sensory processing. Tactile acuity training may normalise sensory processing and improve symptoms. This proof-of-concept trial will assess the feasibility of a novel tactile acuity training method and whether this intervention improves tactile acuity in people with persistent neck pain.

**Methods:**

and analysis: In this two-arm randomised clinical proof-of-concept trial we will recruit participants with neck pain receiving usual care physiotherapy in a secondary outpatient healthcare setting. Thirty-six participants will be randomised 2:1 to receive four weeks of either tactile acuity training using the Imprint Tactile Acuity Device (iTAD) or a placebo intervention, in addition to usual care. The placebo intervention will consist of a de-activated TENS machine (iTENS) said to deliver a sub-threshold inhibitory therapy. Outcomes will be assessed at baseline, mid-treatment, and at 5-weeks and 2-months follow-up. The primary outcome tactile acuity will be evaluated using the two-point discrimination test and locognosia tests. Feasibility will be informed by recruitment and attrition rates, adherence, credibility of the interventions, treatment satisfaction and blinding. Pain intensity and anatomical spread will be analysed as secondary outcomes. The effect of iTAD training on tactile acuity will be assessed using a 2 (Group: iTAD vs. iTENS) x 4 (Time: baseline, mid-treatment, 5-week and 2-month outcome assessment) mixed ANOVA. Secondary outcomes including pain and pain spread, will be analysed with a focus on informing sample size calculations in future trials.

**Ethics and dissemination:**

Risks associated with this study are minor. Usual care is not withheld, and participants consent to random allocation of either iTAD or iTENS. Potential benefits to participants include any benefit associated with the interventions and contributing to research that may assist people with chronic pain in the future. Trial results will be disseminated via academic journals and conference presentations. The study is approved by the Human Research Ethics Committee of Griffith University (2017/128).

## 1 Introduction

Tactile acuity refers to the accuracy and clearness with which we can feel touch. This ability is diminished in several persistent pain conditions [1], including chronic neck pain [2], back pain [3] and knee pain [4]. These sensory changes are thought to reflect changes in central processing that may be part of the aetiology of some persistent pain states [1]. While the substrate of impaired tactile acuity is debated [5], it appears to correspond to changes in primary somatosensory cortex (S1) function in some chronic pain conditions [6,7]. These findings suggest that cortical reorganisation is a shared mechanisms linking reduced tactile acuity to persistent pain [8], although it is not a consistent finding in all persist pain states [9,10].

The finding of tactile acuity deficits in people with persistent neck pain led to the hypothesis that training tactile acuity could improve pain. Indeed, some preliminary studies support this possibility in both neuropathic [11] and non-neuropathic^12 13^ pain conditions, although not all studies report positive effects [14]. Currently, success in reducing pain cannot be directly linked to improved tactile acuity, because tactile acuity is rarely used as an outcome variable [[11], [12], [13], [14]]. Additionally, differences in training paradigms among studies may affect tactile acuity improvements and explain differences in efficacy [14]. A further limitation in current tactile acuity training methods is that they are delivered manually by a clinician or other person over many hours, challenging its practicality [15]. This is especially relevant since previous reports suggest a substantial correlation (r = 0.66, p < 0.05) between time spent training and pain reduction [16].

To make sensory training more feasible, we developed the Imprint Tactile Acuity Device (iTAD)^1721^ that enables training to be carried out independent of a clinician or other person, either at home or in the clinic. Additionally, we designed a customised application that incorporates training parameters linked to successful perceptual learning [[18], [19], [20]]. Our latest data shows that as a test of tactile acuity, the iTAD may have less measurement error relative to the two-point discrimination threshold (TPDT) test [17,21,22]. This indicates that it may be more sensitive to detecting changes over time, such as due to treatment. Moreover, a small cohort study of tactile acuity training in a healthy population supported the possibility that it can improve tactile acuity [21].

While the iTAD has so far shown to be a practical and promising tool to test and train tactile acuity, additional information is needed to design and plan a full-scale RCT in people with chronic neck pain. Particularly, we must confirm that training with the device can improve tactile acuity while establishing the feasibility of an RCT of tactile training in a population with neck pain. Poor recruitment and high attrition rates are common in clinical trials and the substantial patient engagement and participation required may be barriers. Further, information regarding expected changes in pain outcomes are needed to inform sample size calculations.

## 2 Methods

The study is approved by the Human Research Ethics Committee of Griffith University (2016/378) and is currently in registration with the Australia and New Zealand Clinical Trials Register ACTRN 379954. This protocol adheres to the SPIRIT guidelines [23].

### 2.1 Design

**Fig. 1:**
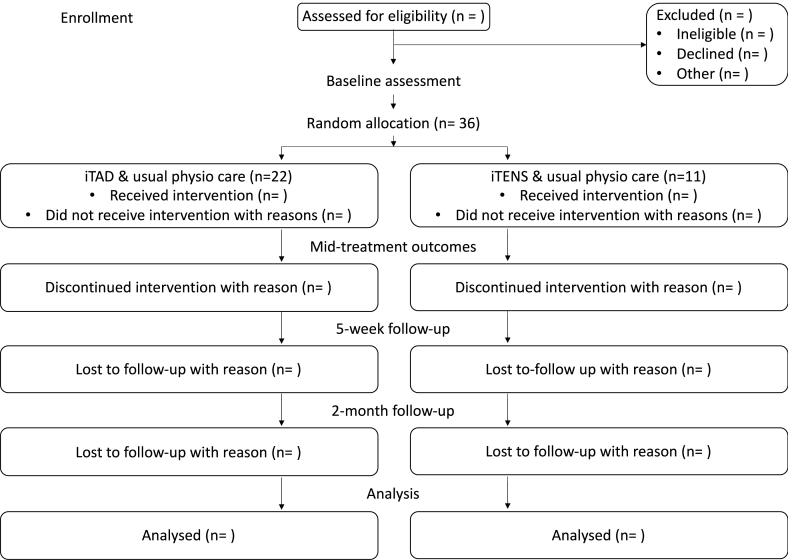
Overview of the flow of participants through the iTAD pilot trial.

A two-arm proof-of-concept randomised clinical trial will be conducted in people with persistent neck pain. All participants will receive usual physiotherapy care with the addition of either iTAD tactile acuity training (experimental group) or iTENS placebo (control group) (see Fig. 1).

### 2.2 Participant selection

Volunteers will be recruited from the Gold Coast University Hospital outpatient physiotherapy clinic. Participants will be considered for the study if aged 18 to 75, have had neck pain of at least 6 months duration and report an average neck pain intensity of at least 3 on a 10-point numeric pain rating scale (NRS) over the past week. Participants will be excluded on the basis of the following criteria: have a diagnosed systemic inflammatory condition (e.g. Rheumatoid Arthritis) or current orthopaedic disorder in their spinal region or upper limbs, a history of neck or head surgery, a history of shoulder surgery in the past 12 months, a history of neurological disease resulting in altered sensation in upper quadrants as indicated by hypoesthesia to touch (unable to feel Von Frey monofilament 4.56 (4.0 g)^24^), presence of neuropathic pain (DN-4 ≥ 4) [24], current self-reported psychiatric condition or psychological disorder other than mild to moderate depression or anxiety. Participants taking medications will not be excluded, however medication use will be recorded for exploratory analyses.

### 2.3 Randomisation and concealment

A concealed stratified permuted block-randomized allocation approach will be employed [25], where participants are randomly allocated to the experimental or control group with a 2:1 ratio. The randomised allocation sequence will be generated using Microsoft excel by an experimenter not involved in data collection. To achieve low allocation predictability, while maintaining the allocation ratio, randomisation to group will occur in blocks of three (2:1) or six (4:2) allocations. The order of the blocks will also be randomised. Further, allocation sequences will be stratified by sex, to ensure equal proportions of males and females in each group. The allocation will be recorded on dark, non-transparent cards, and inserted into opaque envelopes that will be numbered consecutively. Envelopes will be inserted in separate boxes for each sex. After baseline testing, the experimenter will open a sealed envelope, pre-prepared by a third party, containing the participant's allocation.

### 2.4 Blinding

A limited deception approach will be used to maintain participant blinding. While participants understand they will be randomised to one of two groups, one of which may be usual care plus a placebo, once the intervention phase commences the therapist will act as though participants are undergoing active interventions regardless of allocation. Additionally, participants will be blinded towards the allocation ratio. The researcher responsible for assessing outcomes will also be blind to group allocation. A questionnaire with five response categories will be used to quantify blinding of the patient and outcome assessor at the end of the study [26]. Bang's blinding index will be calculated for each treatment arm, as well as for the outcome assessor to estimate blinding success [26]. Patients will also be asked to complete the Credibility Scale to establish the degree to which the iTAD and iTENS interventions were perceived as legitimate [27]. The Credibility Scale will be completed verbally over the phone three days into the intervention.

### 2.5 Sample size

Previous single-armed trials investigating (manual) tactile training for patients with chronic pain found moderate to large effect sizes for within group change in tactile acuity (26% improvement, mean change: TPDT 17.1 mm, p < 0.01, Cohen's d = 1.06) [28] and pain intensity (22% improvement, mean change: VAS 1.0 cm, p = 0.02, Cohen's d = 0.55) [16]. We therefore expect that we need to reach at least medium-large effects in tactile acuity in order to mediate clinically meaningful changes in pain. However, these effect sizes are based on within-group change since a control group was not present. Nevertheless, similar size differences in change of pain intensity have been reported when comparing tactile acuity training to a control condition for: neck pain [29] (24% difference, Δ NRS 1.6 points, p = 0.02, Cohen's d = 0.44); back pain [29] (41% difference, mean change: NRS 2.3 points, p < 0.01, Cohen's d = 1.07); and phantom limb pain [30] (45% improvement, mean change: Multidimensional Pain Inventory 1.5 points, p = 0.02, Cohen's d = 1.16). However, since these studies did not include tactile acuity as an outcome measure, they are not directly informative for our primary research question. As such, a sample size calculation was performed to account for a >90% chance of finding a statistically significant (p < 0.05), medium-large ($\eta_{p}^{2}$ = 0.1) between-group difference in tactile acuity, using a within-between repeated-measures ANOVA with four time-points. The calculation estimated that 18 participants would be required. Accounting for a dropout rate which can be up to 20% for treatments involving active participation in people with health conditions [31,32] a pool of 24 participants (12 in each arm) were estimated to be necessary. To improve power to explore secondary outcomes, particularly within the tactile training group, the target sample size for the intervention arm was doubled (n = 24 intervention group; n = 12 control group).

### 2.6 Usual physiotherapy care

All participants will receive physiotherapy care as usual. The details of usual care received by participants will be recorded. To do this, participants will indicate their co-interventions by selecting from a list of possible types: manual therapy, exercise interventions, home-based exercises, coaching, education, cognitive interventions, pharmacological interventions, invasive interventions, and interventions using applications.

### 2.7 Experimental group

**Fig. 2:**
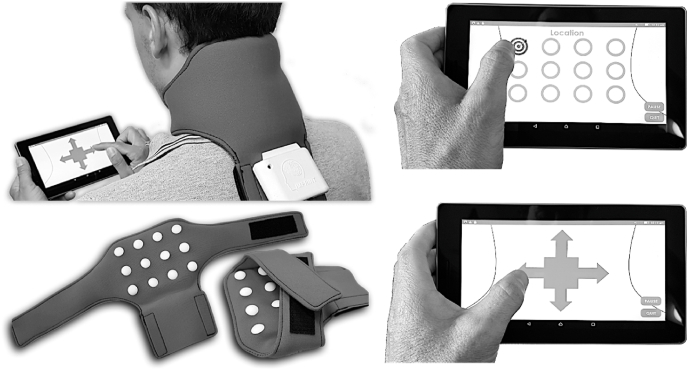
The Imprint Tactile Acuity Device (iTAD) and wirelessly connected tablet displaying the localisation (top right) and orientation (bottom right) applications.

Participants allocated to the experimental group will undergo four weeks of in-home tactile acuity training with the iTAD in addition to their usual physiotherapy care. The iTAD consists of a wearable neoprene collar, containing 12 hemispherical nodes, each enclosing a vibrotactile stimulator (see Fig. 2; a full description has been published previously [17]. The nodes contact the back of the neck and are wirelessly connected to tablet computer with a custom-written software application. The application triggers vibrotactile stimulations, records the user responses, provides feedback and calculates accuracy scores. Training consists of two types of (serious) games: The *localisation game*, where users indicate the location of a single vibration by selecting the corresponding location on the tablet; and the *orientation game*, where users indicate the direction of a second vibration, relative to a first, by selecting the corresponding arrow. The explanation given to participants relating to how iTAD may improve symptoms is standardised (See Appendix 1).

### 2.8 Control group

**Fig. 3:**
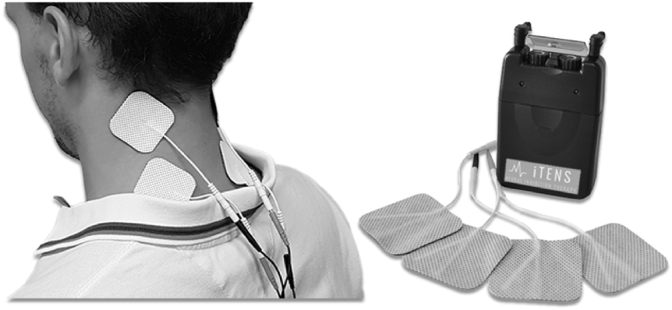
The iTENS machines used to deliver the placebo control intervention, showing the unit, wires and adhesive pads.

Participants in the control group will undergo their usual physiotherapy care plus a placebo intervention. The placebo component of the control intervention is specifically designed to control for non-specific effects of the iTAD. The participants in the control group will undergo four weeks of in-home use of a placebo Transcutaneous Electrical Nerve Stimulation (TENS) unit that will be referred to as iTENS (inhibitory TENS). In order to match time spent with the iTAD, participants will be instructed to use the iTENS for 1 h per day, five days per week. The iTENS devices are altered such that no electrical current is delivered to the user. However, the unit will look fully operational (Fig. 3). The explanation given to participants relating to how iTENS may improve symptoms is standardised (See Appendix 1).

### 2.9 Treatment fidelity and progression

In order to aid adherence and ensure fidelity to protocol, participants will be contacted by the study clinician on a weekly basis during the 4-week intervention and participants will attend a mid-treatment outcome assessment in person. In the experimental group treatment is progressed automatically by increasing the difficulty of the tactile acuity games, the mid-treatment review will be used to implement a false progression of the placebo iTENS machine. Participants will also maintain a treatment log to record frequency and duration of treatment sessions. In the intervention group this will be cross-referenced to the iTAD software usage data for verification.

### 2.10 Primary outcome measures

Tactile acuity measures will be assessed prior to the intervention (baseline), and at mid-treatment, 5-week follow-up (i.e., within 1-week of treatment completion and 5-weeks after commencement), and 2-month follow-up (i.e., 2-months after commencement) (See Table 1). Data relating to feasibility will be collected throughout the study.

**Table 1:** Outcomes measures.

	Domain	Measures	Timepoints
Primary outcomes	Tactile acuity	Two-point discrimination Locognosia	1,2,3,4
			1,2,3,4
	Feasibility	Recruitment rates, adherence (training duration), dropouts Bang's Blinding Index Perceived treatment credibility (Credibility Scale)	Throughout
			4
			*1
Secondary outcomes	Pain Intensity	Pain intensity: 0–10 NRS current, average & worst pain in the past week	1,2,3,4
	Pain spread	Self-indicted on body chart	1,2,3,4
	Tactile acuity	iTAD overall score	1,2,3,4
Exploratory outcomes	Perceived change	Global Perceived Effect (GPE)	2,3,4
	Disability	Neck Disability Index	1,2,3,4
	Tactile acuity	iTAD sub-scores	1,2,3,4
Exploratory predictors	Training hours	Training log	4
	Sleep	Insomnia Severity Index	3
	Psychological	Depression Anxiety & Stress Scale	1
	Baseline measures	Tactile acuity	1

*Time points: 1 = baseline, *1 = three days into the intervention, 2 = mid-treatment, 3 = 5-week outcome assessment (within 1-week of intervention completion), 4 = 2-month outcome assessment.

***Two-point discrimination threshold:*** The participant will be seated with their head resting on their hands on a table in front of them, and a digital sliding two-point Vernier Calliper will be placed under its own weight on the participant's skin to provide two simultaneous tactile stimuli. The calliper will be aligned in a cranio-caudal direction, with the most caudal arm 15 mm lateral to the spinous processes of C7 on the most painful side of the neck. Should pain be bilateral and equal, the side of their dominant hand will be tested. Assessment will commence with the two-points at a 20 mm separation and will be increased by 5 mm increments until the participant report feeling two points of contact. When two distinct points are perceived in three consecutive trials this value will be recorded. The calliper distance will then be increased by a further 10 mm and the same procedure will be performed in reverse, with all following assessments in steps of 2 mm. A series of three ascending and descending assessments will be performed, with the mean of the six values used for analyses. TPDT has excellent intra-rater reliability at the neck (ICC = 0.85, SEM 3.7 mm) [21].

***Locognosia:*** Locognosia, the ability to localise touch, will be assessed with a manual localisation task. Twelve locations (numbered 1–12) will be marked on the neck, in three rows of four, matching the number and inter-node distances (32.5 mm apart in both the vertical and horizontal) of the iTAD. Using the back of the SENSELab brush-05, pressure will be applied to each location three times in a block semi-randomised order. Each block contains one stimulus per location, alternating sides of the neck. Participants will look at a photo of their own neck, with the locations marked, on a tablet and report the number of the location they think is touched. A percentage correct score will be calculated to quantify localisation accuracy. A similar manual locognosia test has demonstrated good (ICC_2.1_ = 0.86) to excellent (ICC_2.1_ = 0.92) intra-rater reliability [33] as well as construct validity using a known-group comparison [33].

***Feasibility:*** Feasibility, with respect to planning a full-scale RCT, will be assessed according to recommended a framework [34]. Data to assess feasibility will be extracted from a digital record of recruitment and screening-related interactions, iTAD digital usage logs, participant completion rates, and questionnaires related to blinding, treatment credibility, and treatment satisfaction. For the protocol to be classified as feasible, a priori cut offs have been determined. These include: 1. A minimum intake of two participants per week and a positive recruitment rate of at least one participant intake out of every four invited, 2. An 80% treatment completion rate, 3. A follow-up rate of 70%, 4. A maximum of 10% missing data for primary and secondary outcomes for those that completed the study, 5. Adherence rates where at least two-thirds of participants complete over 50% of the prescribed training, 6. No statistically significant difference in credibility between intervention and control, a 7. Bangs blinding index of 0 ± 0.2 for both treatment arms as well as the outcome assessor (on a −1 to 1 scale, where scores closer to 0 represents being more unsure of group) [[35], [36], [37]], and 8. Neutral positive satisfaction with treatment ratings for both treatment arms.

***Adverse events:*** For the purpose of this trial, a serious adverse event is defined as an unwanted and harmful outcome that: 1. be related to the intervention, and 2. is significant enough to require medical attention. A minor adverse event will be defined as any unwanted outcome that: 1. either interferes with activities of daily living or persists for more for 24 h, and 2. can be related to the intervention. Adverse events will be monitored during the weekly phone calls with the study clinician.

### 2.11 Secondary outcomes measures

Secondary measures will be assessed prior to the intervention (baseline), and at mid-treatment, 5-week follow-up (i.e., within 1-week of treatment completion and 5-weeks after commencement), and 2-month follow-up (i.e., 2-months after commencement) (see Table 1).

***Pain intensity and pain spread:*** Pain intensity will be assessed using a series of 0–10 NRS, where 0 = no pain, and 10 = worst imaginable pain. Average and worst pain over the past week and current pain will be assessed. A digital tablet (iPad; Apple, Cupertino, CA, USA), a digital stylus (Apple Pencil; Apple, Cupertino, CA, USA) and custom software will be used to obtain the pain drawings and assess pain extent as previously described [38]. The sketching software will display the frontal and dorsal views of the superior half of a body chart and participants will be asked to draw where they feel pain as precisely as possible. The pain extent (% of total body area) is determined by dividing the number of pixels shaded, by total pixels in the body chart areas. Only shaded pixels within the body perimeter are considered. Heat maps will be created to illustrate where pain was most frequently perceived at the four time-points. Heat maps are obtained by overlapping pain drawings from participants in each group, at each time point, and for back and front views.

***iTAD overall tactile acuity score:*** In addition to training tactile acuity, the iTAD also has two tactile acuity tests, measuring localisation and orientation accuracy that can be averaged in to an overall score^17 22^. The tests and procedures have been described previously ^17 22^. Latest data indicates that the overall score has good (ICC_2.1_ = 0.75) inter-rater reliability, with markedly less measurement error than TPDT (coefficient of variation at least 50% smaller) making it more sensitive to change [22].

### 2.12 Exploratory outcomes and predictor variables

Exploratory outcomes are included primarily to inform a future trial and to better understand the iTAD device as a test of tactile acuity. Global perceived effect will be used to explore participant perceived effectiveness of the interventions at each outcome assessment timepoint. Participant will report overall improvement on an 11-point Global Perceived Effect (GPE) scale. The scale spans from −5 (‘much worse’) to 0 (‘no change’) to +5 (‘much better’). Participants who reported a change ≥ +3 at the final outcome assessment will be classified as ‘improved’ [39]. GPE scales are recommended for clinical research [40] and show excellent reliability [41]. The iTAD localisation and orientation sub-scores assessed at each timepoint will provide additional measures of tactile acuity. The Neck Disability Index will be used to explore changes in function [42].

Since the training effect may be affected by factors such as sleep, and psychological status, tactile acuity impairment, and hours training these variables will also be assessed and used as predictors in regression analyses. The Insomnia Severity Scale, Depression, Anxiety and Stress Scale, baseline tactile acuity scores, and training log data will be used to predict change in tactile acuity (for assessment timepoints see Table 1).

### 2.13 Data analysis

The effect of training on tactile acuity will be assessed using a mixed-design two-way analysis of variance (ANOVA) with one between group factor with two levels (Group: iTAD and iTENS) and one repeated factor with four levels (Time: baseline, mid-treatment, 5-week and 2-month outcome assessment). While pain intensity and spread, disability and iTAD scores will be analysed with the same mixed-design AVOVA, the focus of interpretation will be to inform future sample size calculations, and selection of primary outcomes for future trials. For GPE scores, the percentage of people reaching our criterion for improvement (≥+3) will be calculated for each group for descriptive analysis. A multiple linear regression approach will be used to observe whether change in tactile acuity in the iTAD group is predicted by time spent training, baseline tactile acuity scores, baseline sleep (Insomnia Severity Index) and psychological status (Depression Anxiety Stress Scale). Where appropriate, results will be expressed in terms of statistical significance, effect size, descriptive statistics, and plots. Should the final data set contain >5% missing data for the primary outcome, a multiple imputation method will be used to replace missing values [43]. Rates of usual care modalities will be compared between groups in order to identify and discuss their possible influence.

### 2.14 Data management and fidelity to protocol

Participants will be given a unique code at the beginning of the study that will be linked to their personal details (name, contact information, group allocation) via an encrypted digital file. Data will be stored on the research data management cloud platform ResearchSpace using a personal institutional login accessible only by the researchers. Participant information and data will never appear with their name, date of birth, or contact details. In this way, their data are only re-identifiable by the researcher. No identifiable, or re-identifiable, information will be included in notes or publications. Given the short, low-risk, nature of the trial, no independent Data Monitoring Committee is required [44]. Trial conduct will be monitored by members of the team through peer observation of fidelity to protocol, and by peer auditing of data files for entry and transcription errors. Any amendments to the protocol will be submitted to the ethics review board, the Australian New Zealand Clinical Trial Registry, and will be reported in the trial manuscript.

### 2.15 Patient and public involvement

Interviews of people with neck pain attending cross-sectional studies involving the device have been key informants for identifying areas for improvement including: 1. The comfort of the device, 2. The usability of tablet computer user interface, 3. Treatment appeal and likelihood of trial participation, and 4. Instructions for participants. People with neck pain were also consulted regarding the design of the iTENS control condition, and the treatment explanations, and their feedback led to modifications in the language used to ensure its perceived credibility.

## 3 Discussion

### 3.1 Potential impact and significance

Persistent pain conditions represent a massive personal and societal burden [45] Adaptations in the nervous system play a role in many persistent pain states and developing interventions that to target these factors is of high importance [46]. Deficits in tactile acuity demonstrated in people with neck pain and other conditions are likely to reflect a specific type of underlying nervous system adaptation to pain and/or injury [1,2,[47], [48], [49]]. This trial represents an investigation of one of the few treatments specifically designed with these nervous system adaptations in mind. While proposals that training tactile acuity might reduce pain are not new, it has not been widely used in clinical practice because of the time and human resources required to deliver it. The iTAD device has the potential to overcome this barrier, by enabling independent in-home use. This randomised controlled pilot trial is a first step towards establishing whether training with the iTAD can improve tactile acuity and ameliorate symptoms.

### 3.2 Strengths and weaknesses of the study

A strength of the study is the use of participants attending secondary care, a population that may be more likely to present with a significant central nervous system adaptation as a contributor to their symptoms. Further, we have implemented tools to assist in controlling for non-specific effects and bias such as a placebo intervention and blinded assessors. Weaknesses of the study include its inability to blind the therapist and inability to draw conclusions on secondary outcomes due to the low participant numbers in this proof-of-concept study. However, results relating to secondary and exploratory outcomes will assist to inform larger scale studies aiming to determine whether tactile training can improve outcomes such as pain.

## 4 Conclusion

If the current study shows improvement in tactile acuity relative to control, and if secondary data support the possibility of pain reduction, then there are significant potential benefits of continuing iTAD development and research. These include the prospect of a practical method of training tactile acuity and progress towards effective treatments for persistent pain. The trial results will be disseminated via publication in academic journals and presentation at academic conferences.

## Author contributions

Concept/idea/research design: Daniel S. Harvie, Nickile A. Olthof, Michel W. Coppieters, Data collection: Daniel S. Harvie, Andrea Hams, Project management: Daniel S. Harvie, Fund procurement: Daniel S. Harvie, Michel W. Coppieters and Nickile A. Olthof.

## Ethics approval

The study is approved by the Human Research Ethics Committee of Griffith University (2017/128).

## Funding

The study is supported by an Arthritis Australia project grant. DSH is supported by an Early Career Research Fellowship from the 10.13039/501100000925National Health and Medical Research Council of Australia GNT114292.

## Clinical trial registration

The study is registered with the Australia and New Zealand Clinical Trials Register (ACTRN12621000615886). UTN: U1111-1252-9527.

## Declaration of competing interest

The authors declare the following financial interests/personal relationships which may be considered as potential competing interestsAuthors Daniel S Harvie and Nick A Olthof are perusing industry partner grant and commercialisation opportunities for the iTAD.

## Figures and Tables

**Figure:**
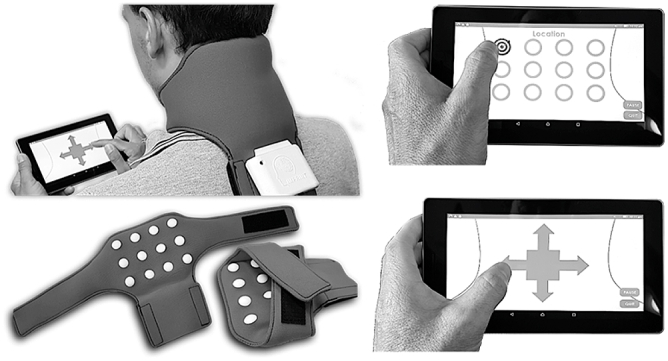


**Figure:**
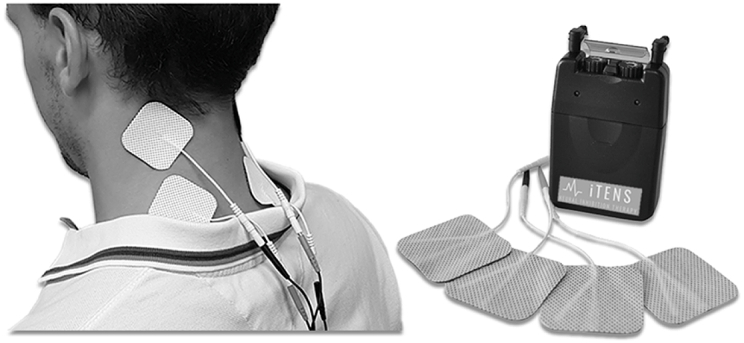


## Appendix 1 iSTOPP study explanations for subjects in the iTAD and iTENS conditions

### The iTAD

The spinal cord and brain continuously filter information coming in from your body. Because information is constantly arriving from all over the body, filtering out unimportant information is essential. In persistent pain, this filtering system is less effective, and pain may be triggered by information that is normally filtered out and ignored. This is much like a bugler alarm being triggered by a moth – not helpful! However, the nervous system can regain its ability to filter information appropriately, and that is what we improve when training with the imprint Tactile Acuity Device (iTAD).

During the treatment, the iTAD will deliver vibrations to the back of your neck and you will have to indicate on the tablet, where you think these vibrations are located. At first, it will be challenging to localise the centre of the vibrations accurately. This is because information that is unimportant to this task will not be filtered out well. With training, you will improve your ability to locate the vibrations, as your nervous system restores its ability to filter information. We anticipate that this will also improve filtering out information that might otherwise lead to unnecessary pain, and this will reduce your symptoms.

### iTENS

The spinal cord and brain continuously filter incoming information from your body. Because information is constantly arriving from all over the body, filtering out (i.e. inhibiting) unimportant information is essential. When pain persists for a while, the nervous system filters less effectively, which can cause pain to arise from information that is normally filtered out. However, the nervous system can regain its ability to filter approprately, and that is what we want to improve with a new type of TENS (Transcutaneous Electrical Nerve Stimulation) called inhibitory TENS (iTENS).

A TENS machine you might already be familiar with, stimulates the nervous system by delivering electrical currents that you can feel. Unlike this type of TENS, the iTENS is designed to de-sensitise the nervous system by delivering the electrical currents below the level that you can feel. Because the current is below what you can feel, the nervous system is tuned down through a process called inhibition. A similar process prevents you being constantly aware of the pressure of your clothes on your skin. We anticipate that this will also improve the nervous system's ability to filter out (inhibit) information that might otherwise lead to unnecessary pain, thereby reducing your symptoms.

## References

[1] Catley M.J., O'Connell N.E., Berryman C. (2014). Is tactile acuity altered in people with chronic pain? A systematic review and meta-analysis. J. Pain.

[2] Harvie D.S., Edmond-Hank G., Smith A.D. (2018). Tactile acuity is reduced in people with chronic neck pain. Musculoskeletal Science and Practice.

[3] Adamczyk W., Luedtke K., Saulicz E. (2018). Lumbar tactile acuity in patients with low back pain and healthy controls. Clin. J. Pain.

[4] Stanton T.R., Lin C.-W.C., Bray H. (2013). Tactile acuity is disrupted in osteoarthritis but is unrelated to disruptions in motor imagery performance. Rheumatology.

[5] Kuroki S., Watanabe J., Nishida S. (2017). Integration of vibrotactile frequency information beyond the mechanoreceptor channel and somatotopy. Sci. Rep..

[6] Flor H., Denke C., Schaefer M. (2001). Effect of sensory discrimination training on cortical reorganisation and phantom limb pain. Lancet.

[7] Pleger B., Dinse H.R., Ragert P. (2001). Shifts in cortical representations predict human discrimination improvement. Proc. Natl. Acad. Sci. Unit. States Am..

[8] Pleger B., Ragert P., Schwenkreis P. (2006). Patterns of cortical reorganization parallel impaired tactile discrimination and pain intensity in complex regional pain syndrome. Neuroimage.

[9] Makin T.R., Scholz J., Filippini N. (2013). Phantom pain is associated with preserved structure and function in the former hand area. Nat. Commun..

[10] Mancini F., Wang A.P., Schira M.M. (2019). Fine-grained mapping of cortical somatotopies in chronic complex regional pain syndrome. J. Neurosci..

[11] Pleger B., Tegenthoff M., Ragert P. (2005). Sensorimotor returning in complex regional pain syndrome parallels pain reduction. Ann. Neurol..

[12] Wand B.M., Abbaszadeh S., Smith A.J. (2013). Acupuncture applied as a sensory discrimination training tool decreases movement-related pain in patients with chronic low back pain more than acupuncture alone: a randomised cross-over experiment. Br. J. Sports Med..

[13] Adriaan Louw P., Kevin Farrell P., Lauren Wettach P.T.D. (2015). Immediate effects of sensory discrimination for chronic low back pain: a case series. N. Z. J. Physiother..

[14] Kälin S., Rausch-Osthoff A.-K., Bauer C.M. (2016). What is the effect of sensory discrimination training on chronic low back pain? A systematic review. BMC Muscoskel. Disord..

[15] Ryan C., Harland N., Drew B.T. (2014). Tactile acuity training for patients with chronic low back pain: a pilot randomised controlled trial. BMC Muscoskel. Disord..

[16] Schmid A.-C., Schwarz A., Gustin S.M. (2017). Pain reduction due to novel sensory-motor training in Complex Regional Pain Syndrome I–A pilot study. Scandinavian journal of pain.

[17] Olthof N.A., Harvie D.S., Henderson C., Thompson B., Sharp R., Craig-Ward L., Coppieters M.W. (2021). Description and psychometric properties of a prototype to test tactile acuity in the neck. Musculoskel. Sci. Pract..

[18] Watanabe T., Sasaki Y. (2015). Perceptual learning: toward a comprehensive theory. Annu. Rev. Psychol..

[19] Maniglia M., Seitz A.R. (2018). Towards a whole brain model of Perceptual Learning. Current opinion in behavioral sciences.

[20] Amitay S., Zhang Y.-X., Jones P.R. (2014). Perceptual learning: top to bottom. Vis. Res..

[21] Harvie D.S., Kelly J., Buckman H. (2017). Tactile acuity testing at the neck: a comparison of methods. Musculoskeletal Science and Practice.

[22] Olthof N., Harvie D., Moseley G. (2021). Modernising tactile acuity assessment; clinimetrics of semi-automated tests and effects of age, sex and anthropometry on performance. PeerJ.

[23] Chan A.-W., Tetzlaff J.M., Altman D.G. (2013). SPIRIT 2013 statement: defining standard protocol items for clinical trials. Ann. Intern. Med..

[24] Timmerman H., Steegers M.A., Huygen F.J. (2017). Investigating the validity of the DN4 in a consecutive population of patients with chronic pain. PloS One.

[25] Broglio K. (2018). Randomization in clinical trials: permuted blocks and stratification. Jama.

[26] Bang H., Flaherty S.P., Kolahi J. (2010). Blinding assessment in clinical trials: a review of statistical methods and a proposal of blinding assessment protocol. Clin. Res. Regul. Aff..

[27] Söchting I., Tsai M., Ogrodniczuk J.S. (2016). Patients' perceptions of treatment credibility and their relation to the outcome of group CBT for depression. Arch. Psychiatr. Psychother..

[28] Gutknecht M., Mannig A., Waldvogel A. (2015). The effect of motor control and tactile acuity training on patients with non-specific low back pain and movement control impairment. J. Bodyw. Mov. Ther..

[29] Hohmann C., Ullrich I., Lauche R. (2012). The benefit of a mechanical needle stimulation pad in patients with chronic neck and lower back pain: two randomized controlled pilot studies. Evid. base Compl. Alternative Med..

[30] Wakolbinger R., Diers M., Hruby L.A. (2018). Home‐based tactile discrimination training reduces phantom limb pain. Pain Pract..

[31] Stubbs B., Vancampfort D., Rosenbaum S. (2016). Dropout from exercise randomized controlled trials among people with depression: a meta-analysis and meta regression. J. Affect. Disord..

[32] Cramer H., Haller H., Dobos G. (2016). A systematic review and meta-analysis estimating the expected dropout rates in randomized controlled trials on yoga interventions. Evid. base Compl. Alternative Med..

[33] Jerosch-Herold C., Rosén B., Shepstone L. (2006). The reliability and validity of the locognosia test after injuries to peripheral nerves in the hand. The Journal of bone and joint surgery British.

[34] Steckler A.B., Linnan L., Israel B. (2002). Process Evaluation for Public Health Interventions and Research.

[35] Houweling A.H., Shapiro S., Cohen J.M. (2014). Blinding strategies in the conduct and reporting of a randomized placebo-controlled device trial. Clin. Trials.

[36] Landsman V., Fillery M., Vernon H. (2018). Sample size calculations for blinding assessment. J. Biopharm. Stat..

[37] Kolahi J., Bang H., Park J. (2009). Towards a proposal for assessment of blinding success in clinical trials: up‐to‐date review. Community Dent. Oral Epidemiol..

[38] Barbero M., Moresi F., Leoni D. (2015). Test–retest reliability of pain extent and pain location using a novel method for pain drawing analysis. Eur. J. Pain.

[39] Thomson H., Evans K., Dearness J. (2019). Identifying psychosocial characteristics that predict outcome to the UPLIFT programme for people with persistent back pain: protocol for a prospective cohort study. BMJ open.

[40] Dworkin R.H., Turk D.C., Farrar J.T. (2005). Core outcome measures for chronic pain clinical trials: IMMPACT recommendations. Pain.

[41] Kamper S.J., Maher C.G., Mackay G. (2009). Global rating of change scales: a review of strengths and weaknesses and considerations for design. J. Man. Manip. Ther..

[42] MacDermid J.C., Walton D.M., Avery S. (2009). Measurement properties of the neck disability index: a systematic review. J. Orthop. Sports Phys. Ther..

[43] Jakobsen J.C., Gluud C., Wetterslev J. (2017). When and how should multiple imputation be used for handling missing data in randomised clinical trials–a practical guide with flowcharts. BMC Med. Res. Methodol..

[44] Sydes M.R., Spiegelhalter D.J., Altman D.G. (2004). Systematic qualitative review of the literature on data monitoring committees for randomized controlled trials. Clin. Trials.

[45] Economics D.A. (2019). The Cost of Pain in Australia.

[46] Moseley G.L., Flor H. (2012). Targeting cortical representations in the treatment of chronic pain a review. Neurorehabilitation Neural Repair.

[47] Zaman J., Vlaeyen J.W., Van Oudenhove L. (2015). Associative fear learning and perceptual discrimination: a perceptual pathway in the development of chronic pain. Neurosci. Biobehav. Rev..

[48] Haggard P., Iannetti G.D., Longo M.R. (2013). Spatial sensory organization and body representation in pain perception. Curr. Biol..

[49] Tsay A., Allen T.J., Proske U. (2015). Sensing the body in chronic pain: a review of psychophysical studies implicating altered body representation. Neurosci. Biobehav. Rev..

